# Protection against Multiple Subtypes of Influenza Viruses by Virus-Like Particle Vaccines Based on a Hemagglutinin Conserved Epitope

**DOI:** 10.1155/2015/901817

**Published:** 2015-02-12

**Authors:** Shaoheng Chen, Dan Zheng, Changgui Li, Wenjie Zhang, Wenting Xu, Xueying Liu, Fang Fang, Ze Chen

**Affiliations:** ^1^Shanghai Institute of Biological Products, Shanghai 200052, China; ^2^National Institutes for Food and Drug Control and WHO Collaborating Center for Standardization and Evaluation of Biologicals, Beijing, China; ^3^Xinhua Hospital Affiliated to Shanghai Jiaotong University of Medicine, Shanghai 200092, China; ^4^College of Life Sciences, Hunan Normal University, Hunan, Changsha 410081, China

## Abstract

We selected the conserved sequence in the stalk region of influenza virus hemagglutinin (HA) trimmer, the long alpha helix (LAH), as the vaccine candidate sequence, and inserted it into the major immunodominant region (MIR) of hepatitis B virus core protein (HBc), and, by using the *E. coli* expression system, we prepared a recombinant protein vaccine LAH-HBc in the form of virus-like particles (VLP). Intranasal immunization of mice with this LAH-HBc VLP plus cholera toxin B subunit with 0.2% of cholera toxin (CTB^*^) adjuvant could effectively elicit humoral and cellular immune responses and protect mice against a lethal challenge of homologous influenza viruses (A/Puerto Rico/8/1934 (PR8) (H1N1)). In addition, passage of the immune sera containing specific antibodies to naïve mice rendered them resistant against a lethal homologous challenge. Immunization with LAH-HBc VLP vaccine plus CTB^*^ adjuvant could also fully protect mice against a lethal challenge of the 2009 pandemic H1N1 influenza virus or the avian H9N2 virus and could partially protect mice against a lethal challenge of the avian H5N1 influenza virus. This study demonstrated that the LAH-HBc VLP vaccine based on a conserved sequence of the HA trimmer stalk region is a promising candidate vaccine for developing a universal influenza vaccine against multiple influenza viruses infections.

## 1. Introduction

Influenza viruses cause acute infections in the respiratory tract. Each year, seasonal influenza results in influenza-related human diseases and deaths around the world. The World Health Organization estimates that yearly human influenza infections are around 1 billion, of which there are 3–5 million serious cases and 300,000–500,000 deaths [1]; and even higher morbidity and mortality occur in pandemic influenza cycles.

Vaccination is an important strategy to prevent and control influenza. But current influenza vaccines are designed for particular influenza strains, which could hardly respond to variations and transmission of influenza viruses. Therefore, there is an urgent need for universal influenza vaccines (UIV) against multiple influenza virus strains, which could quickly and effectively prevent infections and lower transmissions of influenza viruses among human populations at early time. Currently, UIV research has been focused on basic sequences of conserved virus proteins, such as matrix protein 2 (M2) [2] and nucleoprotein (NP) [3]. These experimental vaccines have demonstrated good protection in animal studies, and some have undergone clinical trials. Our team has also used these conserved proteins as vaccine candidate antigens before, such as M2 [4] and NP [5], and explored protection of these sequences in animal models by using multiple vaccine forms such as DNA vaccine [6] and recombinant protein vaccine [4, 5]. In addition, we found that M1 protein also had protective effect [7].

In recent years, one of influenza virus research hotspots was the discovery of many broadly neutralizing antibodies (bNAbs) binding to conserved HA sites (such as CR6261 [8], F10 [9], and CR8020 [10]), and these antibodies displayed good protection in animals and in humans. Meanwhile, progress has been made in UIV research related to these bNAbs and conserved sequences in HA stalk region, such as an optimized HA stalk sequence [11], the HA without its head sequence [12], and the prime-boost immunization strategy [13]. However, in current HA-based UIV research, few of the reported vaccines could elicit robust protective immune responses in animals against lethal viruses challenge or provide cross-protection against different influenza virus strains.

In the present study, we selected a highly conserved long alpha helix (LAH) amino acid sequence in HA2 and used the* E. coli* expression system to express and display this sequence on the surface of hepatitis B virus core (HBc) protein, which formed virus-like particle (VLP) structure. We then tested this LAH-HBc VLP vaccine in the BALB/c mouse model and monitored its immunogenicity and protection against homologous and heterologous influenza virus challenges (including different subtypes of avian influenza viruses), and we preliminarily explored the characteristics of the immune response and the mechanisms of protection.

## 2. Materials and Methods

### 2.1. Viruses and Mice

Influenza viruses used in the experiments were mouse-adapted A/Puerto Rico/8/1934 (H1N1) (GenBank: CY009444.1), A/California/07/2009 (H1N1) (GenBank: KC781785.1), A/Chicken/Jiangsu/7/2002 (H9N2) (GenBank: FJ384759.1), and A/reassortant/NIBRG-14 (Vietnam/1194/2004 x Puerto Rico/8/1934) (H5N1) (GenBank: EF541402.1). All the viruses were frozen at −70°C until use. The whole use of viruses was carried out in a biosafety level 3 containment facility.

Six- to eight-week-old female BALB/c mice (SPF) were purchased from Shanghai SLAC Laboratory Animal Co., Ltd., China. All mice were bred in the Animal Resource Center at Shanghai Institute of Biological Products and maintained in SPF conditions. All experiments involving animals have been approved by Animal Care Committee of Shanghai Institute of Biological Products.

### 2.2. Vector Construction, Expression of Recombinant Target Protein, and Electron Microscopy

The eukaryotic expression vector pCAGGS-P7-HA (PR8 HA) and the prokaryotic expression vector pET28a were kept by Shanghai Institute of Biological Products. Vector 1.3 HBV AF100309 was kindly provided by Shanghai Medicine Molecular Virology Laboratory of Fudan University.

The gene fragments coding for HA2 76–130 amino acids (aa) and HBc 1–149aa were, respectively, amplified from A/PR/8/34 (PR8) HA gene and the genome of hepatitis B virus strain 56 (GenBank: AF100309.1). By overlapping PCR, the former fragment was inserted into the MIR of HBc (replacing 75–85aa), yielding the LAH-HBc gene. Then the LAH-HBc gene was inserted into expression vector, yielding recombinant vector pET28a-LAH-HBc, and the expression was carried out in* E. coli* BL21 (DE3) strain. In short, when the bacteria growth reached the logarithmic phase, 0.5 mM isopropyl *β*-D-1-thiogalactopyranoside (IPTG) was used to induce expression for 6 hours at 28°C. The expression of the target protein was in the form of inclusion body. Through urea denaturation, Ni-NTA purification, dilution renaturation in renaturation solution, dialysis in PBS solution, and ultrafiltration, the inclusion body was purified into recombinant LAH-HBc protein. In addition, the former protein was dialyzed in ultrapure water for desalination.

To obtain direct evidence of VLP formation, samples of the renatured LAH-HBc described above were applied to a plastic/carbon 400-mesh coated grid and incubated for approximately 10 min. Thereafter the grid was stained with 2% sodium phosphotungstate, pH 6.5, for approximately 3 min. The samples were then viewed using a Hitachi H-7000FA transmission electron microscope.

0.2% of CT (Sigma) was added to CTB (Sigma), designated as CTB^*^ with the concentration of 1 *μ*g/*μ*L, used as animal adjuvant.

### 2.3. SDS-PAGE and Western Blotting

The expressed recombinant protein was analyzed for size and purity by SDS-PAGE and western blotting. For SDS-PAGE analysis, the expressed target protein was lysed in loading buffer and then separated by SDS-PAGE, followed by staining with Coomassie brilliant blue R-250. For western blotting, the expressed target protein separated by SDS-PAGE was transferred to PVDF membrane (Millipore). The membrane was blocked and then incubated first with anti-His monoclonal antibody (Novagen) and subsequently with HRP-conjugated secondary anti-mouse antibody (KPL). Binding signals were visualized with TMB substrate.

### 2.4. Immunization and Challenge

Six–eight-week-old female BALB/c mice were anesthetized and immunized three times (2 weeks apart) intranasally with different doses of LAH-HBc protein alone or in combination with adjuvant. The CTB^*^-immunized group was taken as an adjuvant control, and the unimmunized group served as negative control. Three weeks after the last immunization, mice were anesthetized and challenged intranasally with 20 *μ*L of 5 × LD_50_ of A/PR/8/34 (H1N1), A/California/07/2009 (H1N1), A/Chicken/Jiangsu/7/2002 (H9N2), and A/reassortant/NIBRG-14 (Vietnam/1194/2004 x Puerto Rico/8/1934) (H5N1), respectively. Survival rate and weight loss were monitored for 21 days.

### 2.5. Specimens

Three weeks after the last immunization, three mice from each group were randomly chosen for sample collection. The sera were collected from the blood and used for sera IgG assays. Then, their spleens were taken out by sterile forceps to prepare PBMC. Finally, a syringe needle with 1 mL of PBS was inserted three times into the nasopharynx to collect the nasal wash. The nasal wash was centrifuged to remove cellular debris and used for IgA Ab assays.

Three days after the challenge, three mice from each group were randomly chosen for lung collection. The lungs were washed four times by injecting with 2 mL PBS containing 0.1% BSA. After removing cellular debris by centrifugation, the bronchoalveolar wash was packaged into 5 samples, frozen at −80°C, and used for virus titration.

### 2.6. Antibody (Ab) Assays

The titers of IgG and IgA Abs against LAH-HBc recombinant protein were measured by ELISA. ELISA was performed using a series of reagents consisting of, first, 5 *μ*g/mL of LAH-HBc recombinant protein; second, serial 2-fold dilutions of sera or nasal wash from each group of immunized or control mice; third, goat anti-mouse IgG-HRP, IgG2a-HRP, IgG1-HRP, and IgA-HRP (Santa Cruz Biotechnology, Inc.), respectively; and finally, the substrate TMB. The optical density was read at 450 nm. Ab-positive cut-off values were set as means + 2 × SD of preimmunized sera. An ELISA Ab titer was expressed as the highest serum dilution giving a positive reaction.

### 2.7. Passive Serum Transfer

Mice were immunized intranasally with 25 *μ*g LAH-HBc VLP plus CTB^*^ three times with 2-week intervals and their sera were collected at three weeks after last immunization. The negative controls were the sera of PBS treated mice. Mice were infected with a sublethal dose of 0.5 × LD_50_ of PR8 and their sera were collected at three weeks after infection as positive control. Naive mice were given a caudal vein injection of these sera, respectively, and challenged with a lethal dose of 5 × LD_50_ of PR8 within 12 h. Survival rate and weight loss were monitored for 14 days.

### 2.8. Virus Titrations

The bronchoalveolar washing, diluted 10-fold serially, was inoculated to MDCK cells at 37°C for 2 days, so as to examine cytopathic effect. The virus titer of each specimen, expressed as the 50% tissue culture infection dose (TCID50), was calculated by Reed-Muench method. The virus titer in each experimental group was represented by the mean ± SD of the virus titer per mL of specimens from three mice in the group.

### 2.9. IFN-*γ* ELISPOT Assay

Spleen cells were isolated from mice for ELISPOT assays (Dakewe) 3 weeks after the last immunization. According to the instruction manual of Dakewe, 50 *μ*g/mL of LAH-HBc recombinant protein and 50 *μ*g/mL of HA protein (A/California/07/2009 (H1N1)) (NIBSC-UK-EN63QG) were, respectively, used as stimulatory agents. Spots were counted with an ELISPOT reader system (Bioreader 4000; Bio-Sys, Germany). The number of protein-reactive cells was represented as spot forming cells (SFCs) per 10^5^ splenocytes.

### 2.10. Statistics

GraphPad Prism 5 software was used to perform our statistical analyses. The results of lung virus titers and IFN-*γ*-secretion were evaluated by *F*-test (Tukey's multiple comparison test), respectively; the results of body weight change were evaluated by the repeated-measures ANOVA test, respectively; if *P* value was less than 0.05, the difference was considered significant.

## 3. Results

### 3.1. Preparation of Recombinant LAH-HBc VLP

**Figure 1:**
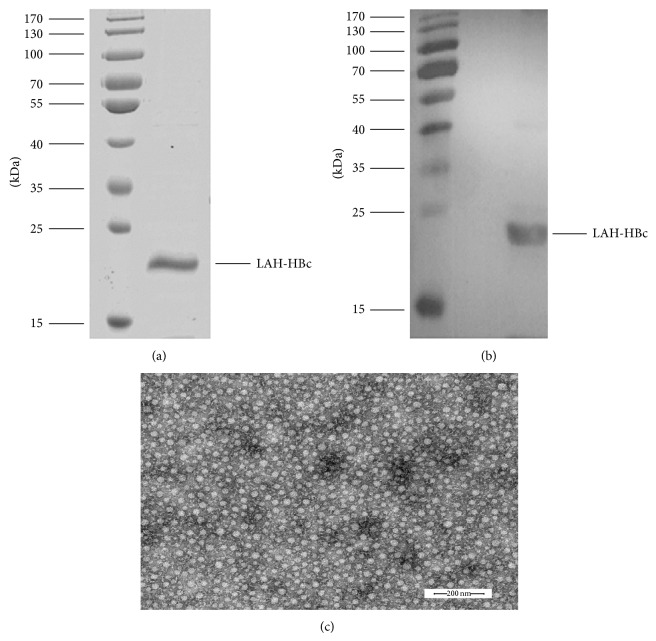
Confirmation of LAH-HBc. (a) Affinity-purified, sterile-filtered LAH-HBc was fractionated by SDS-PAGE under reducing conditions and stained with Coomassie blue. (b) The electrophoresed proteins were transferred to a PVDF membrane on which LAH-HBc was detected using an anti-His mAb. (c) The morphological analysis of LAH-HBc by transmission electron microscopy (TEM).

The gene fragments coding for HA2 76–130aa and HBc 1–149aa were, respectively, amplified from A/PR/8/34 (PR8) HA gene and the genome of hepatitis B virus strain 56, and the former fragment was inserted into the tip of the spike of the major immunodominant region of HBc (replacing 75–85aa), producing the LAH-HBc fusion gene. Then the fusion gene was inserted into a prokaryotic expression vector pET28a and the expression was carried out in* E. coli* BL21 (DE3) strain. The purified recombinant proteins were detected by SDS-PAGE to be a single band at approximately 22 Kda (Figure 1(a)) and further confirmed by western blotting by using anti-His monoclonal antibodies (Figure 1(b)). Electron microscope examination of the purified recombinant proteins revealed that the LAH-HBc proteins were presented in particle form with a diameter of about 30 nm (Figure 1(c)), indicating that the LAH-HBc recombinant protein, expressed by the* E. coli* expression system, could successfully self-assemble into virus-like particles (VLP).

### 3.2. Intranasal Immunization of the LAH-HBc VLP Vaccine Protects Mice against a Lethal Homologous Challenge

**Figure 2:**
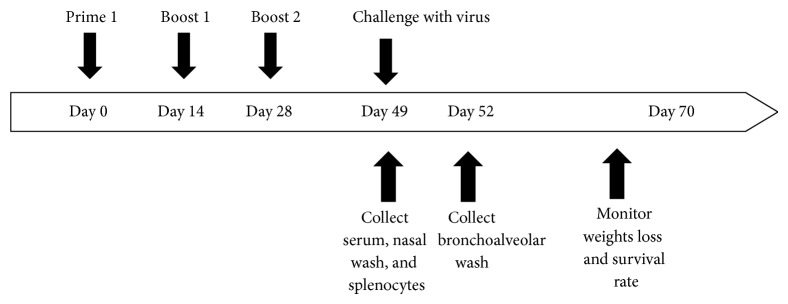
Schematic of the vaccine regimen. BALB/c mice were anesthetized and immunized three times (2 weeks apart) intranasally with different doses of LAH-HBc VLP alone or in combination with CTB^*^ adjuvant. Three weeks after the last immunization, three mice from each group were randomly chosen for sample collection to analyze the adaptive immune response (sera, nasal wash, and splenocytes). Meanwhile, the remaining mice were anesthetized and challenged intranasally with 5 × LD_50_ of influenza viruses. At 3 days after the challenge, 3 mice of each group were randomly taken out for lung lavage and virus titer determination and the remaining 10 mice in each group were observed for survival rates and body weight changes.

Female BALB/c mice were randomly divided into 8 groups of 16 mice each and immunized three times with 2-week intervals (Groups A–H, Table 1). Mice were vaccinated intranasally with 25 *μ*g, 5 *μ*g, and 1 *μ*g LAH-HBc VLP with CTB^*^ adjuvant (Group A, Group C, and Group E) or without CTB^*^ adjuvant (Group B, Group D, and Group F). In addition, there were two control groups, one for adjuvant alone (Group G) and one for blank control (Group H). Three weeks after the last immunization, 3 mice from each group were randomly chosen for sample collection and the rest of the mice were intranasally challenged with 5 × LD_50_ of A/PR/8/34 (H1N1) virus suspension. At 3 days after the challenge, 3 mice of each group were randomly taken out for lung lavage and virus titer determination and the remaining 10 mice in each group were observed for survival rate and body weight change (Figure 2).

**Figure 3:**
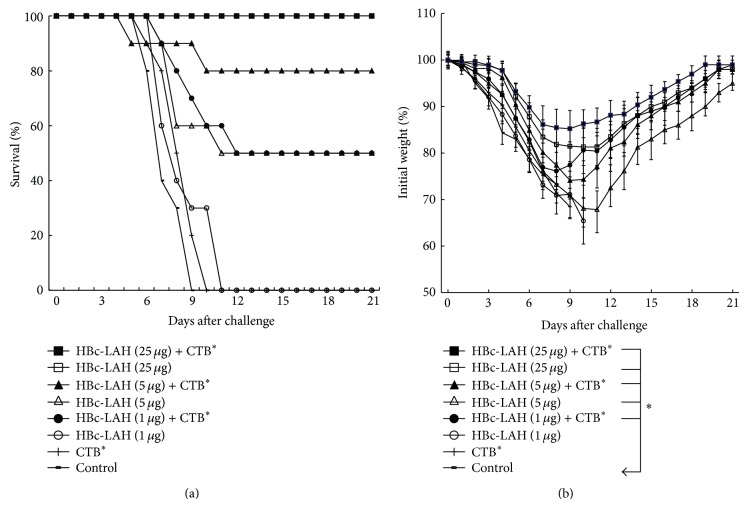
Survival rates (a) and body weight changes (b) after challenge with the mouse-adapted A/PR/8/34 (H1N1) (5 × LD_50_) in mice (10 mice in each group) immunized with LAH-HBc vaccine in combination with or without CTB^*^, three times at an interval of 2 weeks. Mice were challenged 3 weeks after the last immunization. The survival rates and body weights of the mice were measured daily from the date of the challenge to 21 days after the challenge. ^*^Significant difference compared to the control group (*P* < 0.05).

The results showed that homologous protection offered by LAH-HBc VLP vaccine depended on vaccine dose and adjuvant use. As shown in Figure 3(a), both control groups (Groups G and H) had 0% survival, confirming the lethality of the challenge. For vaccination groups without adjuvant, the protection rates of the mice immunized with LAH-HBc VLP alone at dosages of 1 *μ*g (Group F), 5 *μ*g (Group D), and 25 *μ*g (Group B) were 0% (0/10), 50% (5/10), and 100% (10/10), respectively. The result showed that, with the increase of the dosage of  LAH-HBc VLP, the protective effects induced by the vaccine also increased. The protection rates of the mice immunized with 1 *μ*g or 5 *μ*g LAH-HBc VLP plus CTB^*^ adjuvant were 50% and 80%, respectively, while, for the high dose of 25 *μ*g, full protection was achieved with or without adjuvant.

Body weight reduction was observed in all groups (Figure 3(b)). Notably, for the two control groups, the body weight loss was most significant. Meanwhile, the body weight loss of the mice immunized with 25 *μ*g LAH-HBc VLP plus CTB^*^ adjuvant (Group A) was the least, and body weight started to recover 9 days after virus challenge. The body weight loss in other groups was also drastic, and these mice began to recover their body weights 11 days after challenge.

In terms of lung virus titer after challenge (Table 1), all immunization groups had lower titers than the control group, but only the 25 *μ*g LAH-HBc VLP with or without adjuvant groups had significant difference (*P* < 0.05) compared to the control group. Moreover, the lung virus titer had a consistent trend as the protection; that is, the higher the survival, the lower the lung virus titer.

In summary, the above results demonstrated that, as a candidate vaccine, LAH-HBc VLP was capable of protecting mice against a homologous influenza virus challenge. At lower doses of LAH-HBc VLP, adjuvant CTB^*^ could enhance protective effect of the vaccine, but at a high dose of LAH-HBc VLP, full protection against a homologous challenge could be achieved with or without adjuvant.

### 3.3. Antibody Response after LAH-HBc VLP Immunization

As shown in Table 2, for serum IgG level, high level anti-LAH-HBc-specific serum IgG antibody could be detected in all six LAH-HBc VLP immunized groups (Groups A, B, C, D, E, and F), and IgG titer increased with dose of LAH-HBc VLP. No significant difference was found between two groups with the same dose of LAH-HBc VLP with or without adjuvant, indicating CTB^*^ adjuvant has little effect on IgG level after LAH-HBc VLP immunization.

For mucosal IgA level, on the other hand, specific IgA was only found in the 25 *μ*g LAH-HBc VLP with or without CTB^*^ groups and in the 5 *μ*g LAH-HBc VLP with CTB^*^ group (Table 2). IgA titer increased with dose of LAH-HBc VLP; and, for the two groups with the same dose of LAH-HBc VLP (5 or 25 *μ*g) with or without adjuvant, IgA titer was markedly higher in the group with CTB^*^, confirming that the mucosal adjuvant CTB^*^ could enhance local mucosal immune response to LAH-HBc VLP.

In addition, we analyzed the level of IgG1 and IgG2a subtypes of anti-LAH-HBc-specific serum IgG antibody. As shown in Table 3, first, in all immunization groups without CTB^*^, the ratio of IgG2a/IgG1 was greater than 1.0, indicating that the immune response induced was biased toward IgG2a. Second, the immunization groups with CTB^*^ had smaller IgG2a/IgG1 ratio as compared to the same dose protein alone group. This result suggested that LAH-HBc VLP elicited Th1 biased immune response, while LAH-HBc VLP plus CTB^*^ elicited more balanced Th1 and Th2 immune responses.

The above results demonstrated that intranasal immunization of mice with LAH-HBc VLP could not only effectively elicit systemic immune response, but also elicit specific mucosal IgA antibody.

### 3.4. Passive Immune Protection

**Figure 4:**
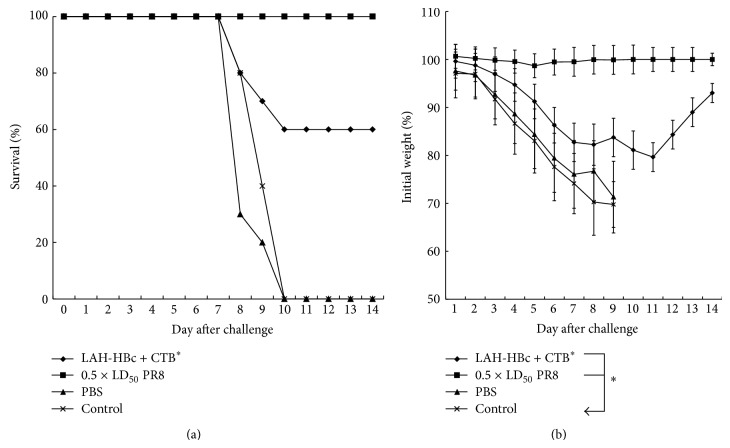
Protection of mice against lethal challenge with homologous virus after pretreatment with anti-LAH-HBc serum. Serum collected from mice immunized with LAH-HBc together with CTB^*^ (designated as LAH-HBc plus CTB^*^ group), from sublethal infection (designated as 0.5 × LD_50 _of PR8 group) or from mice treated with PBS (designated as PBS group), was passively transferred through caudal vein to naïve BALB/c mice (10 mice in each group), respectively. The LAH-HBc plus CTB^*^ group, 0.5 × LD_50 _of PR8 group, PBS group, and blank control group were all challenged with 5 × LD_50_ of PR8 in 12 hours and monitored for survival rates (a) and weight loss (b). ^*^Significant difference compared to the control group (*P* < 0.05).

We carried out passive immunity experiment to evaluate the ability of serum antibodies to provide protection. We collected sera from (1) the group with best protection (25 *μ*g LAH-HBc VLP plus CTB^*^) at three weeks after last immunization, (2) the mice with sublethal infection of 0.5 × LD_50_ of PR8 at three weeks after infection (positive control), and (3) PBS treated BALB/c mice. The sera were, respectively, given to naïve mice, and then within 12 h the mice were challenged with a lethal dose of PR8 (5 × LD_50_). Survival rates and body weight changes were shown in Figure 4. Mice received sera from the PBS group and mice in blank control group displayed quick body weight loss following challenge and all died within 10 days; in contrast, the mice that received the positive control sera (0.5 × LD_50_ of PR8 group) did not display body weight loss and all survived. Mice that received sera from LAH-HBc VLP plus CTB^*^ group displayed a slower body weight decline than the PBS and blank control groups and the final protection rate was 60% (6/10). This passive immunity experiment demonstrated that high titer serum specific antibody induced by LAH-HBc VLP immunization contributed to the protection.

### 3.5. Cell-Mediated Immune Response

**Figure 5:**
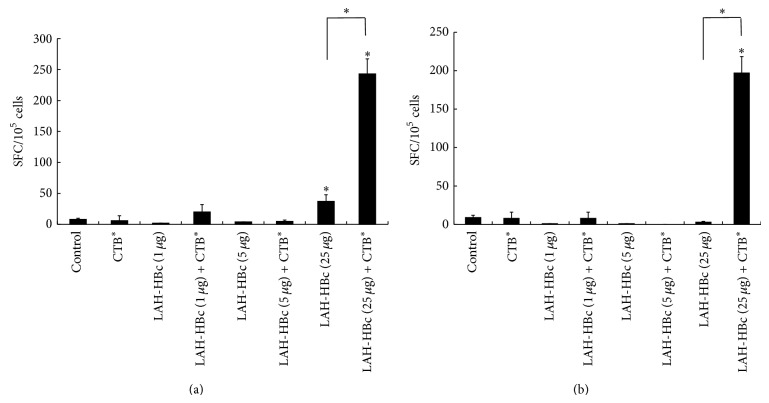
Detection of IFN-*γ* secreted by lymphocyte in spleen. Mice (3 mice in each group) were immunized with LAH-HBc vaccine as described in Table 1. Three weeks after the last immunization, splenocytes were harvested and stimulated with LAH-HBc (a) or HA A/California/07/2009 (H1N1) (b). ^*^Significant difference (*P* < 0.05).

Cell-mediated immune response elicited by the vaccine was evaluated by analyzing specific IFN-*γ*-secreting splenocytes. As shown in Figure 5, first, when stimulated with LAH-HBc VLP, only the two 25 *μ*g LAH-HBc VLP groups had significantly higher IFN-*γ*-secreting spots than the control group, and the group immunized with 25 *μ*g LAH-HBc VLP plus CTB^*^ had significantly more spots than the group immunized with the same dose of LAH-HBc VLP alone (*P* < 0.05). Second, when stimulated with full-length HA protein of A/California/07/2009 (H1N1), specific IFN-*γ*-secreting splenocytes were only detected in one group, the 25 *μ*g LAH-HBc VLP plus CTB^*^ group.

The above results indicated that, after fusing with HBc and assembling into VLP form, the LAH component could effectively elicit specific cellular immune response which could be enhanced by mucosal adjuvant CTB^*^, and this LAH-elicited cellular immune response could specifically respond to the HA of 2009 H1N1 influenza virus.

### 3.6. Intranasal Immunization of the LAH-HBc VLP Vaccine Protects Mice against a Lethal Heterologous Challenge

To determine cross-protection of LAH-HBc VLP against different influenza viruses, we carried out challenge experiments in 25 *μ*g LAH-HBc VLP plus CTB^*^-immunized BALB/c mice with three non-PR8 strains: A/California/07/2009 (H1N1), A/Chicken/Jiangsu/7/2002 (H9N2), and A/reassortant/NIBRG-14 (Vietnam/1194/2004 x Puerto Rico/8/1934) (H5N1), respectively.

**Figure 6:**
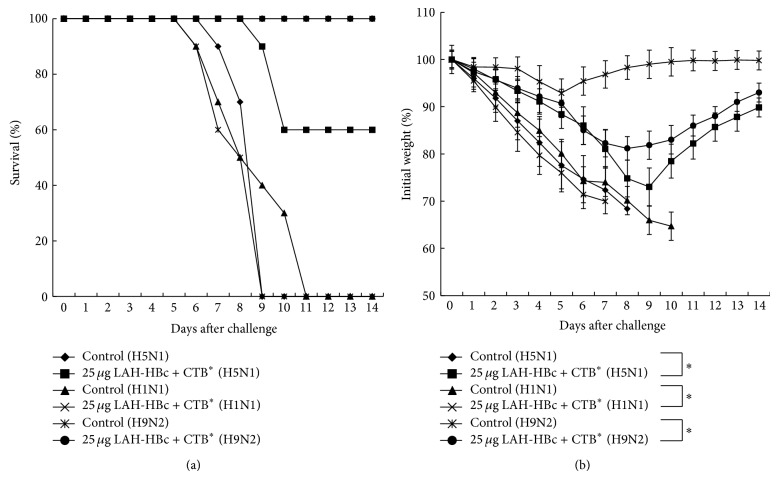
Protection of mice against lethal heterologous influenza A virus challenge by intranasal administration of LAH-HBc with CTB^*^. Sixty BALB/c mice were randomly divided into six groups (10 mice in each group). Three groups were immunized three times with 25 *μ*g LAH-HBc plus CTB^*^ at 2-week intervals. The remaining three groups were unimmunized controls. Three weeks after the last immunization, mice were challenged, respectively, with a lethal dose (5 × LD_50_) of A/California/07/2009 (H1N1), A/reassortant/NIBRG-14 (Vietnam/1194/2004 x PR/8/34) (H5N1), and A/Chicken/Jiangsu/7/2002 (H9N2). The survival rates (a) and bodyweight changes (b) of postinfection were determined. ^*^Significant difference compared to the control group (*P* < 0.05), respectively.

The protection was shown in Figure 6(a). All the immunized mice challenged with a lethal dose of the 2009 H1N1 or the H9N2 virus survived, while only 6 out of 10 immunized mice survived after a lethal challenge of the H5N1 virus with death occurring on day 9 after challenge. All the mice in the nonimmunization control groups died after challenge.

The body weight changes were shown in Figure 6(b). All challenged groups displayed some degree of weight loss following challenge. Mice in the control groups lost weight quickly and then all died. Among the immunized mice, weight loss was the lightest in the group challenged with a lethal dose of 2009 H1N1, with maximal weight loss being 7.2% and weight recovering starting from day 6 following challenge. In contrast, the H5N1 challenge group had the heaviest weight loss, with maximal weight loss being 27.1% and weight recovering starting from day 10 following challenge.

The above results indicated that the LAH-HBc VLP vaccine, which contained HA2 76–130aa of PR8, could not only protect mice from a homologous virus challenge, but also to some degree could cross-protect mice against multiple heterologous viruses. In particular, the cross-protection was robust against infection by the 2009 H1N1 pandemic strain and the avian H9N2 virus.

## 4. Discussion

In recent years, broadly neutralizing antibodies (bNAbs) which neutralized multiple types and subtypes of influenza viruses can be obtained through the new antibody-screening technologies in animal models or in humans. All these bNAbs, such as CR6261, F10, and FI6, target the conserved amino acid sequences in HA and restrict relevant viral functions (such as inhibiting membrane fusion and blocking virus attachment to receptors) so as to achieve broad effect in preventing and treating influenza virus infections.

In 1993, Okuno et al. [14] first reported cross-reactive antibody targeting the stalk of HA trimmer, using A/Okuda/57 (H2N2) virus to immunize mice and homologous virus for screening, and they obtained C179, an antibody which could neutralize both H1 and H2 subtypes of influenza viruses. The discovery of this antibody is of much significance, as it overthrew the traditional understanding of protective antibody of HA. Now this antibody has been commercialized and widely used in research. In recent years, Ekiert et al. [8] identified CR6261, which was obtained through screening memory B cells of humans immunized with seasonal flu vaccines by utilizing phage display technology. Sui et al. [9] identified F10 through screening phage display libraries of unimmunized humans with recombinant H5 trimmer HA protein expressed by insect cells. Subsequently more cross-reactive antibodies targeting HA trimmer stalk region were identified, and their mechanisms of neutralization and protection have increasingly become research focuses.

Wei et al. [13] successfully elicited broad cross-reactive sera in mice, ferrets, and monkeys using H1N1 HA DNA as prime and trivalent seasonal influenza inactivated vaccine or adenovirus vectored HA as boost; these sera could bind all the H1N1 virus strains circulated from 1934 to 2007 and these antibodies were virus neutralizing. In addition, this vaccine could protect mice and ferrets against challenges by different H1N1 viruses at a high lethal dose. Finally, they determined the binding site for these serum antibodies elicited by the vaccine to be HA stalk region and they could compete with C179, an HA stalk-binding antibody discovered earlier. This study was significant in that it was the first study to demonstrate that broadly HA neutralizing antibody targeting HA stalk could be elicited by vaccination.

Meanwhile, the conserved HA sequences targeted by these broadly neutralizing antibodies have started to be studied as candidate target antigens of UIV. As these sequences are located in the interior of HA trimmer and covered by the exposed trimmer head, they are poorly recognized by the immune system, leading to low immunogenicity. Currently two main strategies are taken into account to enhance the immunogenicity of these sequences. The first strategy is to utilize prime-boost strategy with HA DNA vaccine, inactivated vaccine, or virus vectored vaccine [13], and the second strategy is to design an antigen containing the antibody-binding site (such as HA without the trimmer head [12] and conserved amino acid sequence containing HA stalk [15–17]). However, vaccines made by these methods still have poor immunogenicity. They only elicit limited protective immunity in animal models and could not protect mice against a high dose homologous challenge [16, 17], and it was difficult to elicit cross-protection against heterologous influenza viruses.

To improve protective effect of UIV based on conserved HA sequences, many studies were carried out by various researchers and breakthrough progress has been made. Bommakanti et al. [11] used a new method to optimize HA2 sequence and obtained HA2 conformation under neutral pH with the* E. coli* expression system. HA2 expressed with this way had excellent immunogenicity and could bind well with the broad cross-reactive antibody 12D1, and immunized mice could resist a lethal homologous challenge. Bianchi et al. [18] fused the 19 conservative amino acids located at the cleavage site of type B influenza HA0 with the meningococcal envelope protein to increase its immunogenicity. This vaccine could protect mice against challenges of different type B influenza strains. Meanwhile, a vaccine based on HA0 cleavage site of a H3 virus could protect not only against a homologous challenge, but also to some degree against a heterologous H1 virus challenge.

Steel et al. [12] reported that they first removed the head sequence (52–277aa) of HA monomer (HA from PR8 or A/Hong Kong/8/68 (HK68)) and inserted it into a eukaryotic expression vector to construct a DNA vaccine, and then they fused the sequence with HIV gag protein to expression fusion protein VLP vaccine. Then they used DNA prime and VLP boost strategy to immunize mice which could protect mice against a lethal homologous challenge. More importantly, the resulting immune sera had broader cross-reactivity than immune sera obtained with full-length HA immunization. Although the researchers did not explicitly prove that these serum antibodies were specific to the HA stalk region or had neutralizing activity, their data did further confirm the feasibility of UIV based on conserved HA sequences.

Similarly, we made a fusion protein VLP based on conserved HA sequence, but we are the first to utilize the properties of HBc (of displaying exogenous sequence on particle surface and self-assembly into VLP) to make an HBc VLP influenza vaccine based on conserved HA stalk region. We fused HA2 76–130aa (LAH) into HBc and the fusion protein LAH-HBc was successfully expressed and self-assembled into VLP in the* E. coli* expression system. Animal experiments demonstrated that immunogenicity of LAH was significantly enhanced, and immunization in mice elicited high titer serum IgG antibodies which increased with dose of LAH-HBc VLP protein. Meanwhile, in high dose LAH-HBc VLP immunization groups, IgA mucosal antibody could be detected and IgA titer could be significantly enhanced with addition of mucosal adjuvant CTB^*^. These results demonstrated that fusion of HA2 76–130aa (LAH) into HBc could effectively stimulate humoral immune response toward LAH.

Earlier studies showed that HBc [19] as carrier could facilitate the elicitation of cellular immune response toward its surface epitopes. The present study also confirmed that inserting LAH into HBc and adding adjuvant CTB^*^ overcame the low immunogenicity of LAH sequence, and the vaccine demonstrated good immunogenicity and comprehensively elicited systemic humoral immunity, mucosal immunity, and cellular immunity. In addition, intranasal immunization of LAH-HBc VLP provided excellent protection against homologous influenza virus infections.

We found that after intranasal immunization of mice with LAH-HBc VLP, the vaccine with high protection also had high level specific serum and mucosal antibodies. To further test whether specific serum antibody was protective, we carried out the passive protection experiment. The results showed that transferring serum from the group with the highest protection (25 *μ*g LAH-HBc VLP plus CTB^*^) to naïve mice protected 60% of the mice against a lethal homologous (PR8) challenge. This further demonstrated that specific IgG antibodies elicited with LAH-HBc VLP immunization could contribute to protection against virus challenge. Whether these antibodies acted through directly neutralizing virus or through ADCC- or CDC-dependent routes, further studies are needed. In addition, this 25 *μ*g LAH-HBc plus CTB^*^ group also had the highest specific mucosal IgA antibody, and ELISPOT experiments confirmed that this vaccine elicited specific IFN-*γ*-secreting releasing splenocytes response toward the 2009 H1N1 HA. These factors might have accelerated virus clearance after challenge and recovery from infection. Therefore, we drew preliminary conclusion that all the above factors participated in protection of immunized mice against challenge. Given the small sample size and the fact that the study was performed in the mouse model, our initial investigation of the LAH-HBc VLP vaccine is intended as an exploratory study. For further validation on protection efficacy of the LAH-HBc VLP vaccine, further studies should be performed in ferrets or in human clinical trials.

As a segmented RNA virus, the influenza virus easily forms new mutant strains via rapid antigenic drift or shift [20–22]. Once a new mutant strain gains the capacity of efficient human-to-human transmission, it could quickly cause a new round of global pandemic influenza. The 2009 H1N1 global outbreak confirmed this pattern [23, 24]. The present study demonstrated that adjuvanted VLP vaccine based on a conserved HA sequence could well protect mice against a lethal dose infection of the 2009 H1N1 virus, which might be due to high homology between the HA sequence of the vaccine and HA of the 2009 H1N1 virus (as shown in Table 4, the sequence homology was 94.5%).

Since the 1997 [25, 26] Hong Kong outbreak of human infection of highly pathogenic avian influenza H5N1 virus, so far over 400 human infections of avian H5N1 virus have been reported in 14 countries and the mortality was as high as 60%. Human infection of other subtypes of avian influenza viruses, such as H9N2 [27], has also been occasionally reported. The present study demonstrated that the adjuvanted LAH-HBc VLP vaccine could offer some protection to mice against a lethal challenge of H9N2 and H5N1 avian influenza virus (protection was 100% and 60%, resp.). We performed sequence comparison and found that cross-protection after LAH-HBc VLP vaccine immunization was not fully determined by sequence homology between the HA of the vaccine and HA of the challenge virus. As shown in Table 4, the homology between HA2 76–130aa of PR8 virus and that of A/Chicken/Jiangsu/7/2002 (H9N2) was 63.6% (100% protection); the homology between HA2 76–130aa of PR8 virus and that of A/Vietnam/1194/2004 (H5N1) was 81.8% (but the protection was only 60%). We infer that this could be partly explained by the difference of H5N1 and H9N2 viruses in adaption and pathogenicity in mice.

## 5. Conclusions

In conclusion, we prepared the LAH-HBc VLP vaccine, containing a conserved sequence of influenza virus HA stalk region. The fusion protein was expressed by a prokaryotic system in high yield and would be easily scalable with low cost. This vaccine demonstrated good immunogenicity and robust homologous and heterologous protection in a mouse model. Based on results of our study, this LAH-HBc VLP vaccine based on the conserved HA stalk sequence might be an ideal vaccine choice for lowering disease burdens caused by pandemic flu and avian flu and for controlling the circulation of novel influenza virus strains.

## Figures and Tables

**Table 1:** Protection against a lethal PR8 virus challenge in mice by intranasal (i.n.) administration of various doses of LAH-HBc with or without CTB^∗a^.

Group	Immunogen	Immunization route	Dosage (*μ*g)	Protection against PR8 virus challenge	
				Lung virus titers (log_10_TCID_50_/mL)	Survival mice/tested mice
A	LAH-HBc + CTB^*^	i.n.	25	5.92 ± 0.52^b^	10/10
B	LAH-HBc	i.n.	25	6.03 ± 0.38^b^	10/10
C	LAH-HBc + CTB^*^	i.n.	5	6.33 ± 0.35	8/10
D	LAH-HBc	i.n.	5	6.47 ± 0.79	5/10
E	LAH-HBc + CTB^*^	i.n.	1	6.88 ± 1.45	5/10
F	LAH-HBc	i.n.	1	7.35 ± 1.22	0/10
G	CTB^*^	i.n.	—	7.87 ± 0.79	0/10
H	Control	i.n.	—	7.60 ± 0.52	0/10

^a^BALB/c mice were randomly divided into eight groups. Six groups of mice were immunized intranasally with various doses of LAH-HBc vaccine alone or in combination with CTB^*^. The CTB^*^ alone group was used as an adjuvant control, and the unimmunized group served as a negative control. Three weeks after the last immunization, mice were challenged with a lethal dose (5 × LD_50_) of influenza virus (A/PR/8/34 (H1N1)). Bronchoalveolar washes were collected 3 days after infection for titration of lung virus. The survival rate of mice 21 days after infection was determined.

^b^Significant difference compared to the mice in the control group (*P* < 0.05).

**Table 2:** Antibody responses against LAH-HBc in mice induced by intranasal (i.n.) administration of LAH-HBc with or without CTB^∗a^.

Group	Immunogen	Immunization route	Dosage (*μ*g)	Ab responses (ELISA, 2^n^)	
				Serum IgG	Nasal wash IgA
A	LAH-HBc + CTB^*^	i.n.	25	14.67 ± 0.53	6.00 ± 0.43
B	LAH-HBc	i.n.	25	14.33 ± 0.83	3.36 ± 0.78
C	LAH-HBc + CTB^*^	i.n.	5	11.3 ± 0.67	1.30 ± 0.88
D	LAH-HBc	i.n.	5	9.33 ± 0.81	Undetected
E	LAH-HBc + CTB^*^	i.n.	1	8.00 ± 0.95	Undetected
F	LAH-HBc	i.n.	1	7.03 ± 0.43	Undetected
G	CTB^*^	i.n.	—	Undetected	Undetected
H	Control	i.n.	—	—	—

^a^Mice were immunized with LAH-HBc vaccine with or without CTB^*^ as described in Table 1. The serum samples and nasal washes were examined by ELISA for specific IgG and IgA Abs, respectively.

Results are expressed as means ± SD of tested mice in each group.

**Table 3:** IgG1 and IgG2a antibody responses against LAH-HBc in mice induced by intranasal (i.n.) administration of LAH-HBc with or without CTB^∗a^.

Group	Immunogen	Immunization route	Dosage (*μ*g)	Serum Ab responses (ELISA, 2^n^)		
				IgG1	IgG2a	IgG2a/IgG1
A	LAH-HBc + CTB^*^	i.n.	25	11.00 ± 1.00	12.00 ± 1.00	2
B	LAH-HBc	i.n.	25	11.00 ± 1.15	13.00 ± 1.58	4
C	LAH-HBc + CTB^*^	i.n.	5	9.33 ± 1.48	9.33 ± 1.04	1
D	LAH-HBc	i.n.	5	7.00 ± 0	8.67 ± 1.07	3.18
E	LAH-HBc + CTB^*^	i.n.	1	7.00 ± 0	7.67 ± 1.15	1.59
F	LAH-HBc	i.n.	1	6.33 ± 0.58	7.33 ± 0.58	1.99
G	CTB^*^	i.n.	—	Undetected	Undetected	—
H	Control	i.n.	—	—	—	—

^a^Mice were immunized with LAH-HBc vaccine as described in Table 1. The serum samples were examined by ELISA for specific IgG1 and IgG2a, respectively.

Results are expressed as means ± SD of tested mice in each group.

**Table 4:** Sequence identities of the HA2 76-130aa between A/PR/8/34 (H1N1) and the tested heterologous influenza A virus.

Influenza strain	Identities	Survival mice/tested mice	The maximum rate of weight loss
A/California/07/2009 (H1N1)	52/55 (94.5%)	10/10 (100%)	7.2%
A/Vietnam/1194/2004 (H5N1)	45/55 (81.8%)	6/10 (60%)	27.1%
A/Chicken/Jiangsu/7/2002 (H9N2)	35/55 (63.6%)	10/10 (100%)	18.8%
